# Supplementary material for: A new mouse mutant with a discrete mutation in Pcdhgc5 reveals that the Protocadherin γC5 isoform is not essential for dendrite arborization in the cerebral cortex
Source: PLoS One. 2026 Mar 12;21(3):e0344863. doi: 10.1371/journal.pone.0344863 (PMC12981507; doi:10.1371/journal.pone.0344863)

high mw transfer

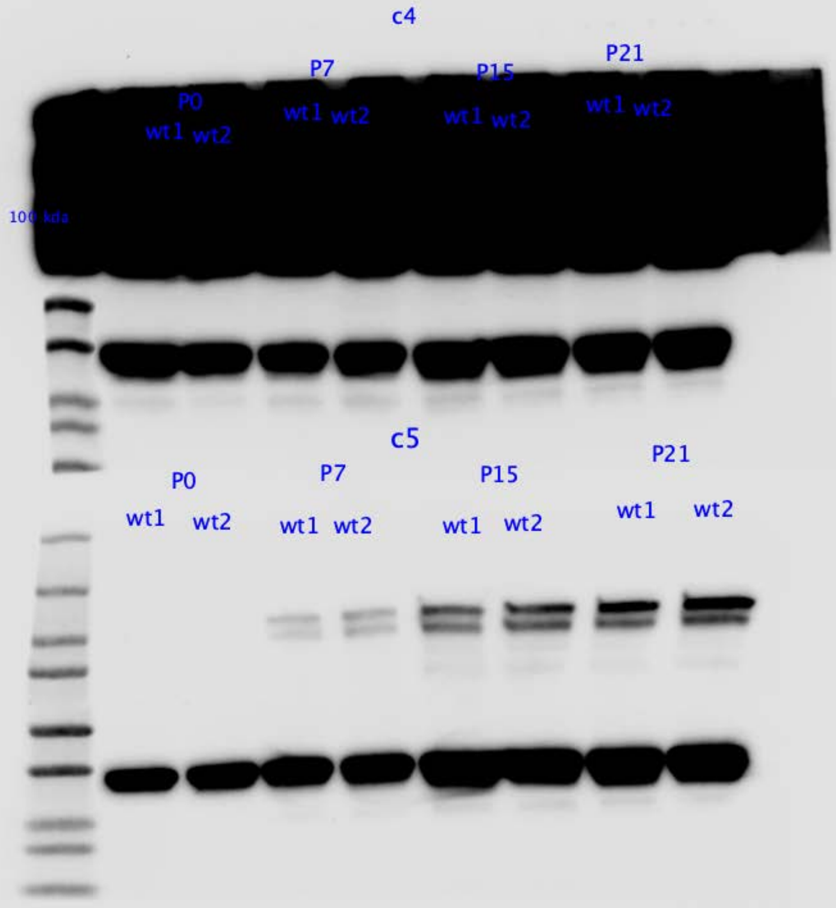

high mw transfer

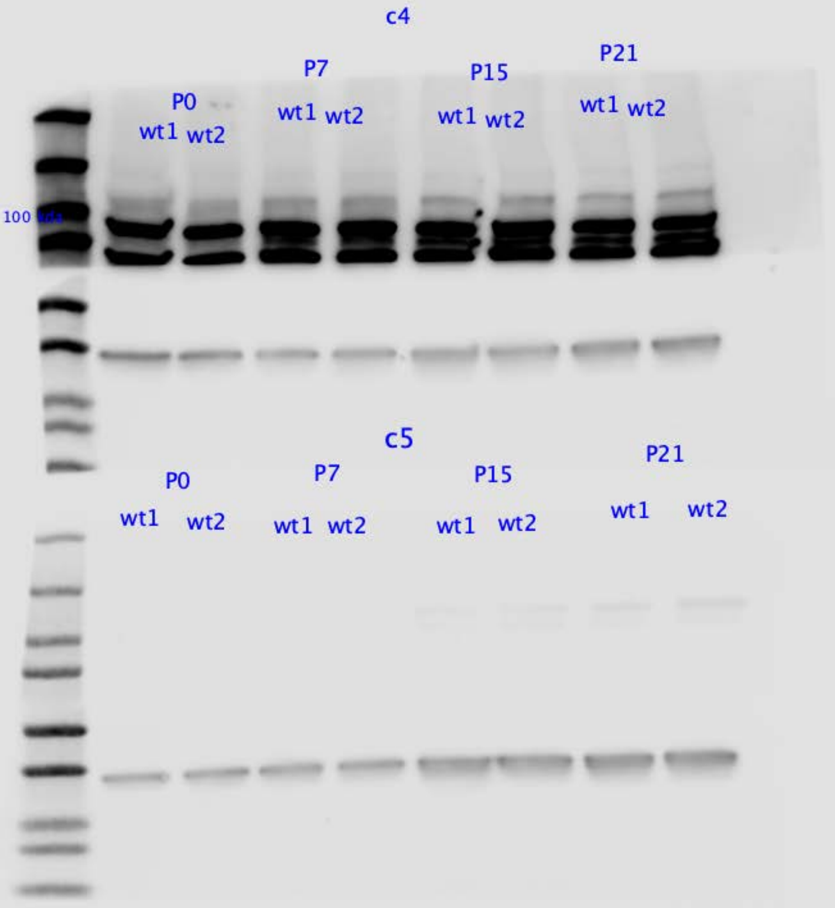

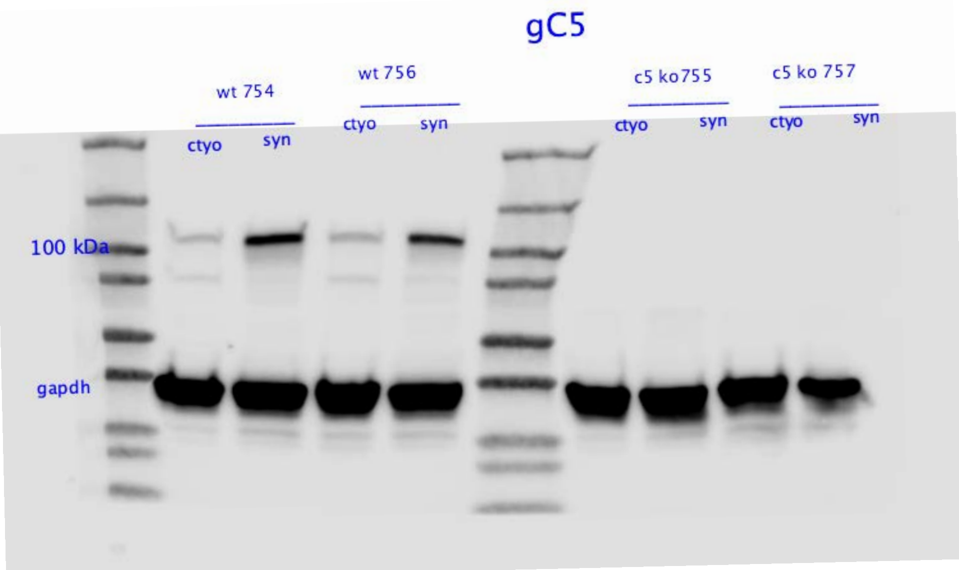
gC5
wt 754
wt 756
c5 ko755
c5 ko 757
ctyo
syn
ctyo
syn
ctyo
syn
ctyo
syn
100 kDa
gapdh

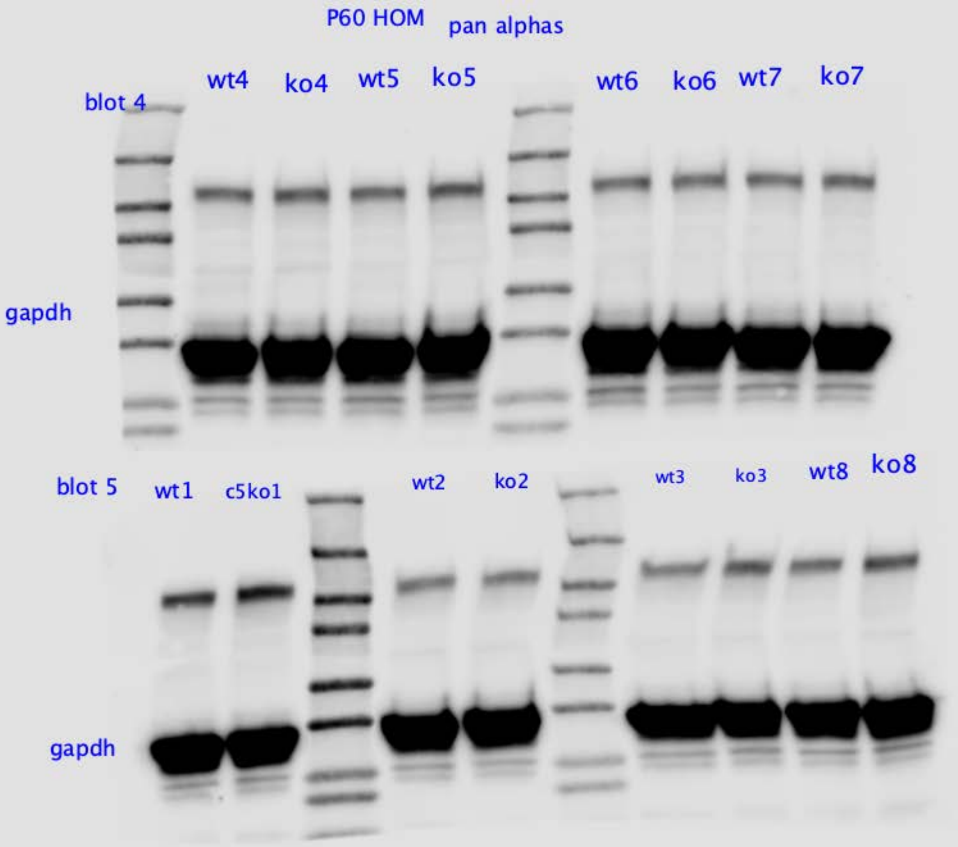
P60 HOM
pan alphas
blot 4
wt4
ko4
wt5
ko5
wt6
ko6
wt7
ko7
gapdh
blot 5
wt1
c5ko1
wt2
ko2
wt3
ko3
wt8
ko8
gapdh

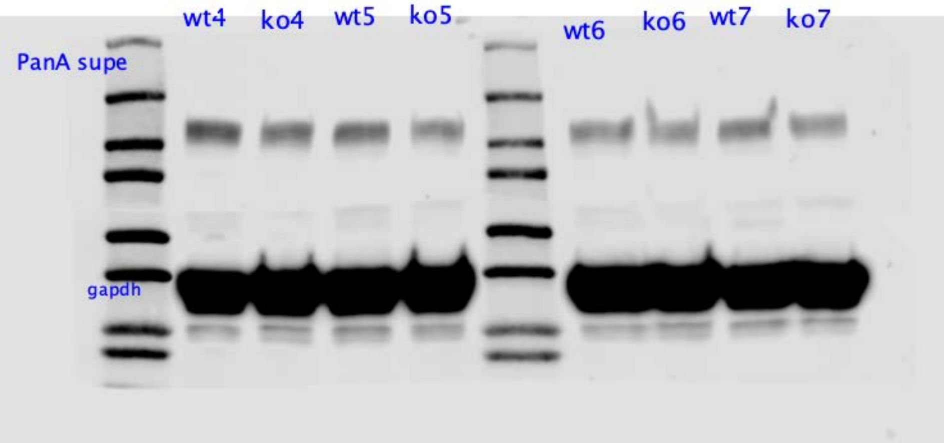
wt4
ko4
wt5
ko5
wt6
ko6
wt7
ko7
PanA supe
gapdh

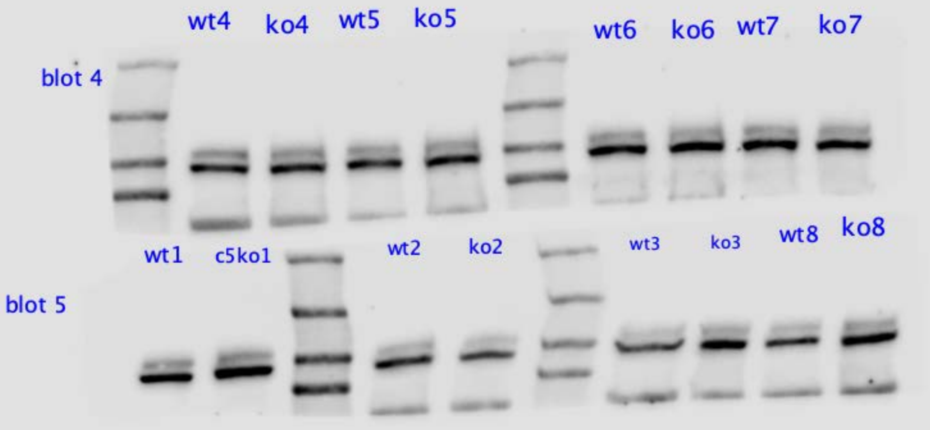
wt4
ko4
wt5
ko5
wt6
ko6
wt7
ko7
blot 4
wt1
c5ko1
wt2
ko2
wt3
ko3
wt8
ko8
blot 5

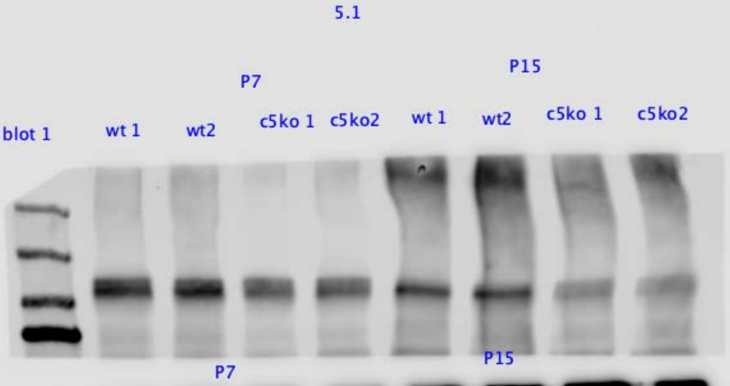
5.1
P7
P15
blot 1
wt 1
wt2
c5ko 1
c5ko2
wt 1
wt2
c5ko 1
c5ko2
P7
P15

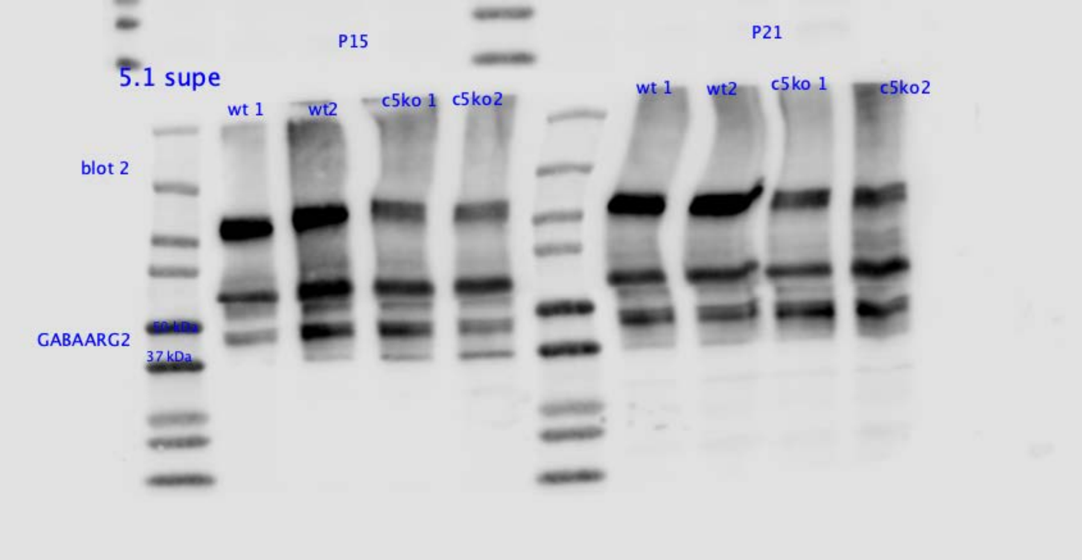
5.1 supe
P15
P21
wt 1
wt2
c5ko 1
c5ko2
wt 1
wt2
c5ko 1
c5ko2
blot 2
GABAARG2
50 kDa
37 kDa

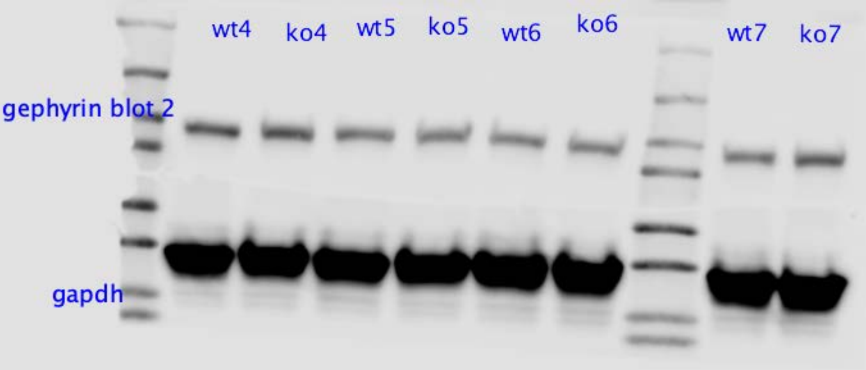
wt4
ko4
wt5
ko5
wt6
ko6
wt7
ko7
gephyrin blot 2
gapdh

P60 PSD95

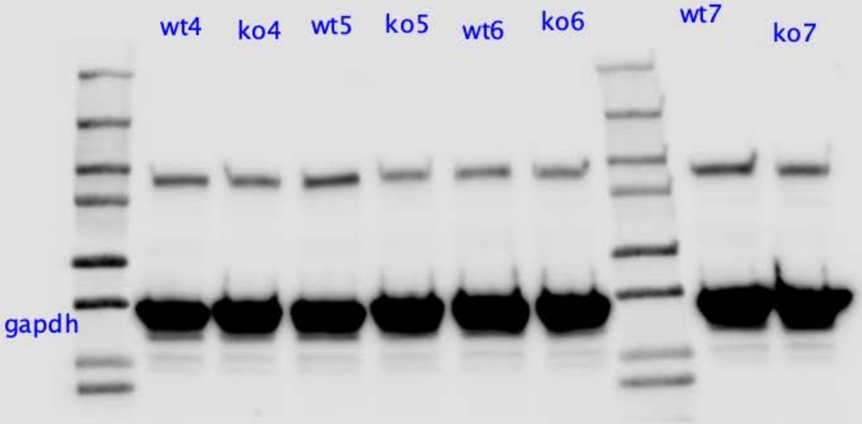

geph blot stripped and reprobed with synapsin
P60 HOM

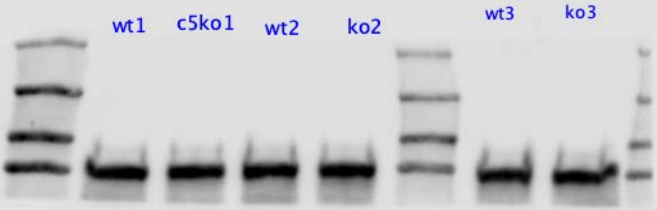

Supplement: S1 Raw Images — (PDF) [file pone.0344863.s001.pdf]
