# Supplementary material for: A new mouse mutant with a discrete mutation in Pcdhgc5 reveals that the Protocadherin γC5 isoform is not essential for dendrite arborization in the cerebral cortex
Source: PLoS One. 2026 Mar 12;21(3):e0344863. doi: 10.1371/journal.pone.0344863 (PMC12981507; doi:10.1371/journal.pone.0344863)
Supplement: Attachment — Submitted filename: Response to Reviewers.docx [file pone.0344863.s004.docx]

We thank the editor and reviewer for their comments on the manuscript.

The reviewer raised two points:

“Here are my two minor concerns.

1. The RNA-seq analysis highlights alterations in pathways related to synaptic activity, learning/memory, and circadian regulation. While this is interesting, no functional assays (e.g., behavioral tests or electrophysiology) were included that might link these transcriptomic observations to physiological outcomes.

2. The transcriptomic profiling was conducted at a single postnatal timepoint, which may not reflect earlier, transient developmental effects, especially given the known timing of γC5 expression onset. Additionally, several differentially expressed genes were not independently validated.

Clarifying these points or adding a brief discussion would strengthen the conclusion.”

We have addressed these points by adding text to the discussion. We agree that the RNA-seq analysis would be bolstered by future studies with functional analyses and finer validation, including a time course to capture developmental effects. However, these analyses are outside the scope of the current study. Our main goals here were to describe the new mouse mutant targeting the γC5 isoform and to test this isoform’s role in dendrite arborization. As our findings conflict with a recent study that found deficient dendrite arborization in a different mouse purporting to target γC5, we put emphasis on the expression of other clustered protocadherin isoforms, and the RNA-seq data were an important component of this.

Other updates include formatting changes requested by the editor (e.g., removing funding information from the acknowledgements), adding the GEO accession information for the RNA-seq data, and minor corrections to Figure 2 (addition of a label to 2F, correction of asterisks in 2B). We have also included raw Western blots and an excel file with every datapoint.
